# Risk factors for nutrition-related chronic disease among adults in Indonesia

**DOI:** 10.1371/journal.pone.0221927

**Published:** 2019-08-30

**Authors:** Vanessa M. Oddo, Masumi Maehara, Doddy Izwardy, Anung Sugihantono, Pungkas B. Ali, Jee Hyun Rah

**Affiliations:** 1 University of Washington School of Public Health, Department of Health Services, Seattle, Washington, United States of America; 2 Child Survival and Development, United Nations Children’s Fund, Jakarta, Indonesia; 3 National Institute of Health Research and Development, Ministry of Health, Jakarta, Indonesia; 4 Disease Prevention and Control, Ministry of Health, Jakarta, Indonesia; 5 Public Health and Nutrition, Ministry of National Development Planning, Jakarta, Indonesia; University of Cape Coast, GHANA

## Abstract

**Objective:**

To conduct a secondary data analysis detailing the associations between sociodemographic and behavioral factors and nutrition-related chronic disease.

**Methods:**

These analyses utilized 2014 data from the Indonesian Family Life Survey, a home-based survey that collected socioeconomic, dietary intake, physical activity, and biological data among adults. We explored four outcomes in relation to sociodemographic and behavioral determinants: 1) hypertension, 2) elevated high-sensitivity c-reactive protein (hs-CRP), and 3) central obesity, as these are critical metabolic determinants in the progression to cardiovascular disease, and 4) type 2 diabetes. Hypertension was defined as systolic blood pressure ≥140 mm or diastolic blood pressure ≥ 90mm or current use of antihypertensive medication. Elevated hs-CRP was defined as hs-CRP >3 mg/dL. Central obesity was defined as waist circumference ≥ 90 cm if male and waist circumference ≥ 80 cm if female, which are specific to South Asia. Type 2 diabetes was defined as glycated hemoglobin ≥ 6.5%. We employed separate gender-stratified multivariate logistic regression models to test the associations between sociodemographic and behavioral determinants and each nutrition-related chronic disease outcome. All analyses employed sampling weights, which account for the survey design.

**Results:**

In 2014, about 30% of adults were hypertensive and one-fifth had elevated hs-CRP. Approximately 70% of women had central obesity and 11.6% of women and 8.9% of men had diabetes. Older-age was consistently associated with nutrition-related chronic disease and being overweight was associated with hypertension, elevated hs-CRP, and type 2 diabetes. Regularly consuming instant noodles (women) and soda (men) were associated with elevated hs-CRP and soda consumption was associated with central obesity among men.

**Conclusions:**

Large segments of the adult population in Indonesia now have or are at risk for non-communicable disease. Our analyses provide preliminary empirical evidence that interventions that target healthful food intake (e.g. reduce the intake of ultra-processed foods) should be considered and that the reduction of overweight is critical for preventing chronic diseases in Indonesia.

## Introduction

Non-communicable diseases (NCDs) have become the leading causes of death in middle-income countries [1]. In Indonesia nearly three-quarters of all deaths are attributed to NCDs, of which one-third are due to cardiovascular disease (CVD) [2]. Indonesia is also home to 10 million diabetic individuals, which ranks sixth in the world [3] and the prevalence is greater than 10% in remote, non-urban areas [4]. While cancer is less prevalent in Indonesia, about 350,000 new cases of cancer are diagnosed each year, accounting for 12% of mortality in 2016 [2].

Concurrently, the leading causes of disability adjusted life years in Indonesia were heart disease, cerebrovascular disease, and type 2 diabetes, in 2016 [5]. This high NCD burden is a major driver of health care spending in Indonesia. In 2015, healthcare expenditures totaled USD 28 million [6], which translates into USD 383 annually per person, more than half of which are individual out-of-pocket expenditures [7]. By 2040 health expenditures are expected to triple, largely due to the increasing NCD prevalence [7]. In addition, between 2012 and 2030, the projected economic output loss due to NCDs is an estimated USD 4.5 trillion [8]. Moreover, NCDs are thought to be a major barrier to achieving the Sustainable Development Goals.

The nutrition transition, characterized by unhealthier diets and physical inactivity, has played a major role in the behavioral and metabolic risk factors for NCDs. Unhealthy diets, particularly diets high in fat [9], sodium [10], and sugar [11–13], have been strongly associated with a range of NCDs. Dietary risk factors, including high intake of sodium and low intake of whole grains and fruits, are among the leading causes of NCD-related deaths and disability in Indonesia and globally [5,14]. Diet data in Indonesia, particularly as it relates to the consumption of energy- or sodium-dense foods, is limited. However, available data have shown inadequate consumption of fruits and vegetables, high intake of sodium, and an increase in the percent of total energy coming from fat [3,14–19]. Physical inactivity has also contributed to NCDs [20] and disability, in Indonesia [5], and data suggest that the population is increasingly adopting a more sedentary lifestyle [16,21–23].

In turn, a number of studies have linked these behavioral risk factors to metabolic risk factors for NCDs [9,24–27]. For example, excess sodium consumption is related to hypertension risk [24,25], and excess sugar consumption is related to central obesity [26] and elevated high sensitivity-C-reactive protein (hs-CRP) [28,29], all of which are related to CVD [19,30,31]. Hypertension and high body mass index (BMI) were the top metabolic risk factors driving death and disability in Indonesia in 2017 [5]. Relatedly, between 1993 and 2007, central obesity and hypertension increased by 22% and 7%, respectively [21].

Relatively few studies have used national data to explore both sociodemographic and behavioral determinants of nutrition-related NCDs in Indonesia. Fewer have used biological data to do so. The primary aim of this paper was to conduct a secondary data analysis detailing four outcomes in relation to sociodemographic and behavioral determinants: 1) hypertension, 2) elevated hs-CRP, and 3) central obesity, as these are critical metabolic determinants in the progression to CVD, and 4) type 2 diabetes. We believe this evidence will be useful in informing policies and programs that aim to reduce NCDs in Indonesia [21].

## Materials and methods

### Survey design and study population

These analyses utilized the Indonesian Family Life Survey (IFLS), an ongoing longitudinal, home-based study that was initiated in 1993 [32]. There have been four subsequent survey rounds (1997, 2000, 2007, 2014). The original, multi-stage sampling frame was based on households from 13 out of 27 provinces, which represented 83% of the Indonesian population in 1993 [32]. For these analyses, we utilized 2014 data from adults aged above 19 years, due to data availability (described below). In Indonesia, eight new provinces have been created since 1999, thus, in 2014, 24 of 34 provinces were represented.

Among the original 33,081 household members enrolled in the IFLS, about one-third, (11,040) were found in their original IFLS households in 2014, approximately 9,000 were found elsewhere and about 4,500 had died. The recontact rate (including deaths) in 2014 among individuals enrolled in 1993 was 76%. Over the course of the survey, 11,889 (54%) responded in all survey waves [32].

### Survey questions & measurements

Data were collected on household- and individual-level characteristics, as well as diet, physical activity, and health. The household questionnaire was completed by the head of household and recorded information on household size, physical infrastructure, access to sanitation, area of residence (urban/rural), and food expenditures. While the topics covered in the individual-level adult questionnaire were wide-ranging, these analyses utilized information on gender, age, educational attainment, marital status, employment, and smoking status. Adults were asked whether they ate each food type “in the last week” and as appropriate, the frequency of consumption (number of days). Self-reported consumption of dietary staples (e.g. rice, eggs, meat, green leafy vegetables, sweet potatoes) was recorded, and in 2014, consumption of some ultra-processed foods, including instant noodles, fast food, soft drinks, and fried snacks, were also recorded. Ultra-processed foods are typically “ready-to-consume” and are entirely or mostly made from industrial ingredients and additives, not foods [33]. In addition to being energy-dense, they are also characteristically high in fat, sugar, and/or salt (unlike rice, which would be considered energy-dense). Consumption of ultra-processed foods was of interest in these analyses as they have become more common worldwide [33], their availability and consumption increases as countries undergo their nutrition transition, and their consumption has been associated with poor chronic-disease related health [34–37] and weight gain in one experimental study [37].

Adults in this sample also self-reported any vigorous physical activity, moderate physical activity, and/or walking (during the last week) using a modified version of the International Survey on Physical Activities. Vigorous activity was defined as any activities that make you breathe much harder than normal (e.g. heavy lifting). Moderate activity was defined as any activities that make you breathe somewhat harder than normal (e.g. carrying light loads). Walking included walking from place to place, walking for recreation, and walking at work and at home.

All health measurements were collected by trained enumerators, using a rigorous research protocol, whereby measurements were supervised and subject to quality control procedures [32]. Height was measured to the nearest millimeter using a Seca plastic height board. Weight was measured to the nearest one-tenth of a kilogram using a Camry model EB1003 scale. Waist circumference (for adults aged ≥ 40 years) was measured to the nearest millimeter with a tape measure. Blood pressure was taken three times, on alternate arms from a seated position, using an Omron meter, HEM-720. Large cuffs were available as needed.

In 2007 (IFLS wave 4), dried blood spots (DBS) were collected among a random sample of individuals from wave 1 of the IFLS (1993). Those same respondents were re-contacted in 2014 to continue to collect DBS. A finger prick was taken and blood drops drawn for measurement of hs-CRP and glycated hemoglobin (HbA_1c_). Prior to the finger prick, hand warmers were used to increase blood flow. The first drops of blood were used with the hemoglobin and secondary drops were put onto Whatman 903 Protein Saver Cards [32]. Cards were allowed to dry for at least 4 hours and stored with a desiccant to keep samples dry. Samples were kept cool, mailed back to headquarters in Yogyakarta, and then, stored at—40 Celsius, until assayed [38]. The assay used to measure hs-CRP in 2007 was no longer on the market when the most recent IFLS wave was fielded [38]; in 2014, the hs-CRP enzyme immunoassay kit was manufactured by Percipio Biosciences (Catalog Number 11190) [39]. The HbA_1c_ assay was based on a validated protocol, described by Hu and colleagues [40]. Validation samples, for both hs-CRP and HbA_1C_, were provided by the University of Washington (Seattle, U.S.) and the University of Southern California/University of California Los Angeles’ Center on Biodemography and Population Health (Los Angeles, U.S.) prepared additional blood spots that were used as controls for the assays [39]. Samples were analyzed at the University of Washington and in Indonesia. Using regression-based methods, DBS results were converted to plasma-equivalent values for hs-CRP and whole blood equivalent values for HbA_1c_, based on repeated measurements of the validation samples [39]. Approximately, 6,300 adults aged ≥ 19 years (~ 2,800 men and ~ 3,500 women) had usable CRP or HbA_1c_ data and sample weights. Subjects with incomplete data and pregnant women were excluded from these analyses.

### Statistical analysis

We explored associations between risk factors and four dependent variables: hypertension, elevated hs-CRP, central obesity, and type 2 diabetes (henceforth referred to as diabetes). Systolic and diastolic blood pressure were based on the average of three measurements. Hypertension was defined as systolic blood pressure ≥140 mm or diastolic blood pressure ≥ 90 mm or current use of antihypertensive medication [41,42]. Elevated hs-CRP was defined as hs-CRP >3 mg/dL, based on increased risk for CVD [31]. Central obesity was defined as waist circumference ≥ 90 cm if male and waist circumference ≥ 80 cm if female, which are specific to populations in South Asia [43]. Clinical guidelines define diabetes as glycated hemoglobin (HbA_1C_) ≥ 6.5% [44].

Prior literature, along with the availability of data in the IFLS, were used to identify the independent variables in regression models. Consumption of ultra-processed foods (versus not consuming), physical activity (versus no activity), smoking (versus not smoking) and urban residence (versus rural) were modeled as binary variables. Appropriate cutoffs were applied to create dichotomous or categorical variables for age, education, employment, family size, and food expenditures. A composite of household wealth was created using principal component analysis, using following variables: type of floor material, type of toilet, type of cooking fuel and ownership of assets including: land, livestock, vehicle(s), household appliances, furniture and utensils, jewelry, and monetary savings. Wealth was divided into quintiles, based on the distribution of the data. In models exploring hypertension, elevated hs-CRP, and diabetes as the outcome, overweight/obesity (henceforth referred to as overweight) was included as an independent variable and was defined as BMI ≥ 25 kg/m^2^.

We first employed separate, gender-stratified univariate logistic regression models to investigate risk factors and their association with each outcome (S1–S4 Tables). Independent variables that were statistically significantly associated with the dependent variable in univariate models were then included in multivariable logistic regression models. Age, urban/rural residence, and wealth were included in the multivariable models irrespective of statistical significance.

In sensitivity analyses we defined overweight as BMI ≥ 23 kg/m^2^, because a substantial proportion of Asian people have a higher risk of diabetes and CVD at lower BMIs [45] (S5–S7 Tables). We assessed multicollinearity in all models. Alpha was set to 0.05. All analyses employed sampling weights, which account both for sample attrition from 1993 to 2014, and account for the original sample design, which included an over-sample of urban areas; thus, this sample is representative of the 2014 Indonesian population in those provinces. Analyses were performed using Stata 15.1 (StataCorp LP, College Station, TX). Survey participants provided written informed consent. The data used for this study are retrospective and the authors did not have access to any identifying information. As such, ethical approval was not required.

## Results

A majority of the sample (N = 15,648 women, N = 13,567 men) was middle-aged (30–59 years) and about half had a primary level of education (Table 1). Nearly all women were non-smokers (96.3%), compared to 32.8% of men. About one-third of women were not working (37.1%), compared to 10.2% of men. Half of the adults resided in urban areas and slightly larger proportions resided in lower (versus higher) wealth households.

**Table 1:** Selected characteristics of adults in Indonesia, 2014.

	Women		Men	
	N = 15,648		N = 13,567	
	n	%^a^	n	%^a^
Demographic and Sociodemographic				
Age (in years)				
19–29	4,350	16.0	3,397	23.0
30–39	4,458	18.3	3,990	18.9
40–49	2,827	24.7	2,774	19.1
50–59	2,074	21.6	1,755	20.8
≥ 60	1,939	19.5	1,651	18.1
Education				
No Education	1,078	13.3	389	5.4
Primary	5,026	47.3	4,197	44.7
Junior or Senior	4,955	30.1	4,738	38.4
University	1,618	9.2	1,471	11.5
Marital Status				
Never Married	1,288	5.2	2,174	14.4
Married	12,112	74.3	10,808	80.4
Other	2,244	20.5	580	5.1
Employment Status				
Not working	5,876	37.1	1,198	10.2
Agriculture-based Labor	2,397	19.6	3,532	29.4
Skilled Manual Labor^b^	1,335	8.9	2,852	21.3
Skilled Labor^c^	5,771	34.3	5,744	39.2
Residence				
Rural	6,439	51.0	5,596	49.8
Urban	9,209	49.0	7,969	50.2
Wealth				
Lowest	2,903	24.7	2,857	26.1
Second	2,628	22.0	2,500	22.0
Middle	1,887	16.8	1,826	16.8
Fourth	2,191	18.6	2,077	18.4
Highest	2,058	17.8	1,930	16.8
Province				
Bali	780	2.2	711	2.1
Central Java	2,008	17.4	1,705	17.2
East Java	2,152	26.0	1,774	23.5
Jakarta	936	5.1	854	5.1
Lampung	632	3.3	603	3.5
North Sumatra	1,128	5.0	1,014	5.1
South Kalimantan	702	3.1	607	2.9
South Sumatra	732	3.6	698	4.0
South Sulawesi	797	3.6	635	2.9
West Java	2,141	17.9	1,812	21.3
West Nusa Tenggara	1,183	3.1	986	2.7
West Sumatra	748	3.2	600	3.0
Yogyakarta	812	6.6	752	6.6
Food Expenditures				
Rice Lowest	8,184	55.1	7,058	54.3
Rice Highest	7,442	44.9	6,469	45.7
Cooking Oil Lowest	9,404	61.6	8,257	62.0
Cooking Oil Highest	6,220	38.4	5,268	38.0
Mean Family Size				
≤ 4	9,846	64.9	8,471	63.3
> 4	5,802	35.1	5,094	36.7
Behavioral Risk Factors				
Smoking Status				
Does Not Smoke	15,119	96.3	4,421	32.8
Currently Smoking	447	3.7	9,077	67.2
Physical Activity (Last Week)^d^				
Vigorous Activity	1,558	10.8	4,668	38.0
Moderate Activity	8,696	58.1	6,844	54.1
Walking	10,051	68.8	9,298	72.4
Diet (Last Week)				
Consumed Instant Noodle	9,567	59.8	8,479	63.4
Consumed Fast Food	1,647	8.9	1,246	8.7
Consumed Soda	1,859	10.6	3,153	22.2
Consumed Fried Snacks	9,472	65.3	8,483	68.8
Health Indicators				
Hypertension^e^	4,168	35.8	3,472	29.9
Central Obesity^f^	4,555	66.2	1,575	23.9
Type 2 Diabetes^g^	315	11.6	226	8.9
Elevated hs-CRP^h^	774	21.4	403	14.9
Comorbid Conditions^i^	2,947	60.0	1,908	36.4
Medicine for Hypertension^j^	575	5.1	274	2.5
Medicine for Type 2 Diabetes^j^	194	1.7	142	1.3

hs-CRP = high sensitivity c-reactive protein

^a^ Percentages are weighted to be representative of the population of Indonesia during a given survey year. Missings: education (women n = 2,971, men n = 2,772); marital status (women n = 4, men n = 5); employment (women n = 269, men n = 241); residence (women n = 0, men n = 2); wealth (women n = 3,981, men n = 2,377); province (women n = 897, men n = 816); food expenditures rice (women n = 22, men n = 40); food expenditures oil (women n = 25, men n = 42); family size (women n = 0, men n = 2); smoking (women n = 82, men n = 69).

^b^ Skilled manual labor combines the following employment sectors: mining, manufacturing, electric, gas, water maintenance, and construction

^c^ Skilled labor combines the following employment sectors: retail and service, transportation

^d^ Defined using the International Physical Activity Questionnaire

^e^ Systolic and diastolic blood pressure are based on the average of three measurements. Hypertension is defined as systolic blood pressure ≥140 mm or diastolic blood pressure ≥ 90 mm or current use of antihypertensive medication.

^f^ Measured only among adults ≥ 40 years old. Specific to populations in South Asia: central obesity is defined as waist circumference ≥ 90 cm if male and waist circumference ≥ 80 cm if female

^g^ Defined as HbA_1C_ ≥ 6.5%

^h^ Defined as hs-CRP > 3 mg/dL

^i^ Comorbidity is defined as the coexistence of any two (or more) of the following conditions: hypertension, central obesity, type 2 diabetes, elevated hs-CRP

^j^ Self-reported medication use

Approximately 60–70% of adults reported regularly consuming instant noodles and fried snacks. Approximately one-quarter of men regularly consumed soda, but relatively few adults consumed fast food (< 10%). About 40% of men were vigorously active, compared to only 10.8% of women. A majority of adults were moderately active (~55%). Approximately 30% of men and women were hypertensive. Women had a higher prevalence of elevated hs-CRP (women: 21.4%, men: 14.9%), central obesity (women: 66.2%, men: 23.9%) and diabetes (women: 11.6%, men: 8.9%). Among women, 60.0% had two (or more) of the following conditions: hypertension, central obesity, type 2 diabetes, elevated hs-CRP. Comorbidities were also prevalent among men (36.4%). Few adults (≤5%) reported taking medication for hypertension or diabetes.

**Fig 1:**
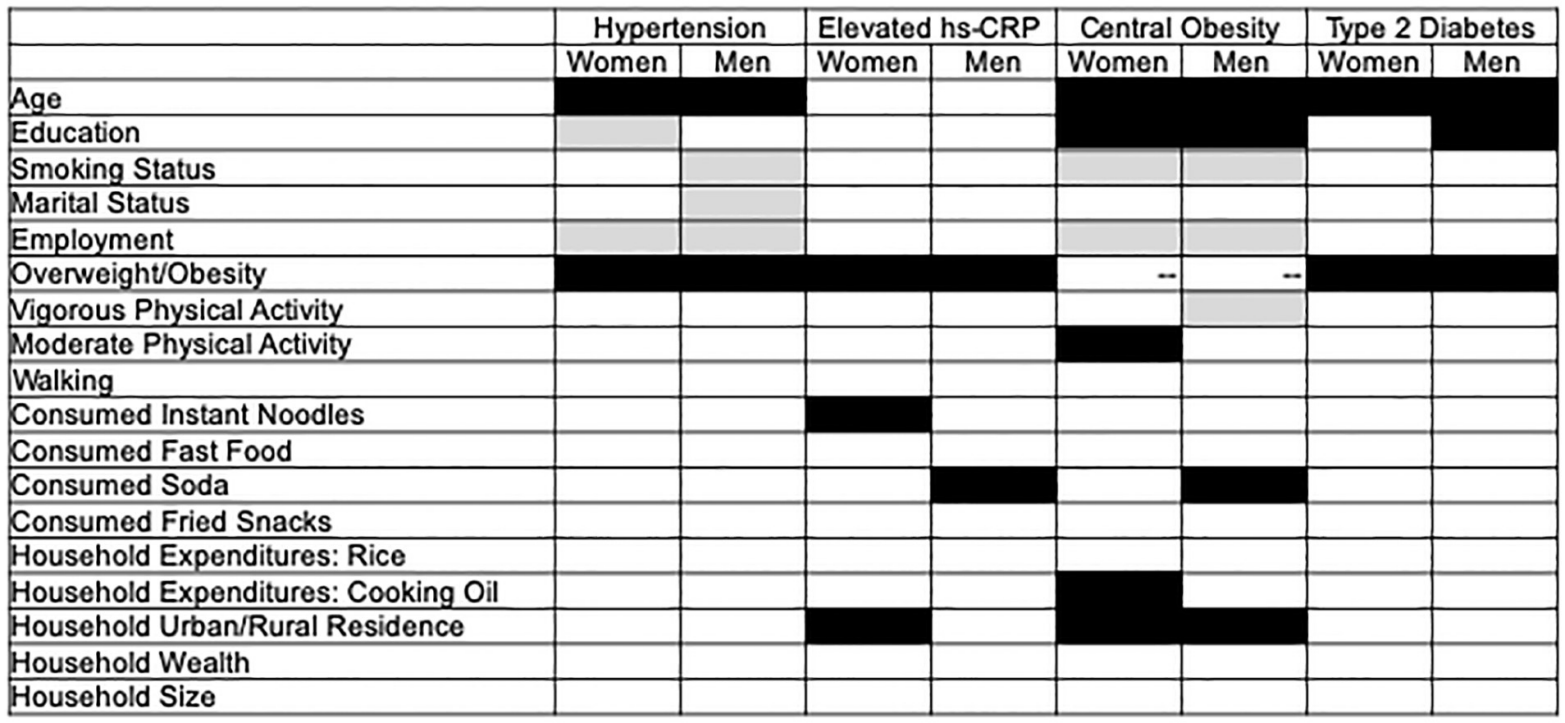
Summary of associations between selected characteristics and chronic disease among adults in multivariable models. --not applicable. **Associated with higher odds of chronic disease outcome**. Associated with lower odds of chronic disease outcome.

Fig 1 summarizes all associations between hypothesized risk factors and chronic disease-related outcomes in multivariable models.

### Hypertension

Among women, older age and being overweight were associated with higher odds of hypertension (Table 2). Conversely, higher educational attainment (Odds Ratio [OR] = 0.67; 95% Confidence Interval (CI): 0.48, 0.94) was associated with lower odds of hypertension. Being employed in agriculture-based labor (OR = 0.66; 95% CI: 0.54, 0.80) or skilled labor were also associated with lower odds of hypertension (OR = 0.72; 95% CI: 0.62, 0.84).

**Table 2:** Multivariable Logistic Regression Testing the association between selected characteristics and hypertension among adults in Indonesia, 2014.

	Women^a^^,^^b^	Men^a^^,^^b^
	N = 8,854	N = 8,745
Individual Level	Odds Ratio (95% CI)	Odds Ratio (95% CI)
Age (in years)		
19–29	Reference	Reference
30–39	2.65 (2.06, 3.41) *	1.19 (0.89, 1.59)
40–49	6.48 (5.02, 8.35) *	2.69 (2.00, 3.62) *
50–59	13.94 (10.64, 18.26) *	4.45 (3.28, 6.04) *
≥ 60	23.05 (16.99, 31.28) *	9.48 (6.87, 13.09) *
Education		
No Education	Reference	
Primary	1.09 (0.85, 1.39)	1.04 (0.72, 1.50)
Junior or Senior	0.86 (0.66, 1.13)	0.94 (0.63, 1.39)
University	0.67 (0.48, 0.94) *	1.04 (0.66, 1.62)
Marital Status		
Never Married	Reference	Reference
Married	1.13 (0.70, 1.84)	0.75 (0.54, 1.04)
Other	1.22 (0.72, 2.09)	1.00 (0.59, 1.72)
Employment		
Not Working	Reference	Reference
Agriculture-based Labor	0.66 (0.54, 0.80) *	0.65 (0.48, 0.87) *
Skilled Manual Labor^c^	0.81 (0.63, 1.03)	0.71 (0.52, 0.97) *
Skilled Labor^d^	0.72 (0.62, 0.84) *	0.70 (0.53, 0.94) *
Overweight (BMI ≥ 25 kg/m^2^)		
No	Reference	Reference
Yes	2.11 (1.85, 2.40) *	2.77 (2.33, 3.30) *
Smoking Status		
Does not smoke	Reference	Reference
Currently Smoking	1.08 (0.75, 1.57)	0.82 (0.70, 0.96) *
Physical Activity (Last Week)^e^:		
No Vigorous Activity		Reference
Vigorous Activity	---	0.83 (0.71, 0.98) *
No Moderate Activity	Reference	Reference
Moderate Activity	0.94 (0.82, 1.07)	0.92 (0.79, 1.07)
Consumed (Last Week):		
Instant Noodles		
No	Reference	Reference
Yes	0.98 (0.85, 1.12)	1.01 (0.86, 1.18)
Fast Food		
No	Reference	Reference
Yes	1.09 (0.87, 1.38)	0.97 (0.73, 1.29)
Soda		
No	Reference	Reference
Yes	0.94 (0.76, 1.16)	1.03 (0.85, 1.25)
Household Level		
Residence		
Rural	Reference	Reference
Urban	1.05 (0.92, 1.21)	1.00 (0.85, 1.18)
Wealth		
Lowest	Reference	Reference
Second	0.96 (0.79, 1.15)	0.82 (0.66, 1.01)
Middle	0.99 (0.81, 1.21)	0.90 (0.71, 1.13)
Fourth	0.96 (0.79, 1.16)	0.84 (0.67, 1.06)
Highest	0.89 (0.73, 1.09)	0.80 (0.64, 1.02)
Family Size		
≤ 4	Reference	
> 4	0.92 (0.81, 1.05)	---

BMI = body mass index, CI = confidence interval

^a^ Systolic and diastolic blood pressure are based on the average of three measurements. Hypertension is defined as systolic blood pressure ≥140 mm or diastolic blood pressure ≥ 90 mm or current use of antihypertensive medication

^b^ Odds ratios and confidence intervals are estimated using logistic regression and are weighted to account for the survey design. Models exclude women who are currently pregnant.

^c^ Skilled manual labor combines the following employment sectors: mining, manufacturing, electric, gas, water maintenance, and construction

^d^ Skilled labor combines the following employment sectors: retail and service, transportation

^e^ Defined using the International Physical Activity Questionnaire

* *p* < 0.05

Men who were aged ≥ 40 years (versus 19–29 years old) had an approximately 3- to 9-fold higher odds of hypertension. Being overweight (OR = 2.77; 95% CI: 2.33, 3.30) was associated with higher odds of hypertension. Smoking (versus not smoking) (OR = 0.82; 95% CI: 0.70, 0.96) was associated with lower odds of hypertension among men. Being employed in agriculture-based labor (OR = 0.65; 95% CI: 0.48, 0.87), skilled manual labor (OR = 0.71; 95% CI: 0.52, 0.97) or skilled labor (OR = 0.70; 95% CI: 0.53, 0.94) were also associated with lower odds of hypertension.

### Elevated hs-CRP

Overweight (OR = 2.42; 95% CI: 1.67, 3.51) and urban (versus rural) residence (OR = 1.51; 95% CI: 1.00, 2.31) predicted elevated hs-CRP among women (Table 3). Every additional day women consumed instant noodles was associated with 12% higher odds of elevated hs-CRP (OR = 1.12; 95% CI: 1.01, 1.25).

**Table 3:** Multivariable Logistic Regression Testing the association between selected characteristics and elevated hs-CRP among adults in Indonesia, 2014.

	Women^a^^,^^b^	Men^a^^,^^b^
	N = 1,055	N = 453
Individual Level	Odds Ratio (95% CI)	Odds Ratio (95% CI)
Age (in years)		
19–29	---	Reference
30–39	---	1.30 (0.51, 3.29)
40–49	---	1.18 (0.43, 3.23)
50–59	---	0.58 (0.15, 2.30)
≥ 60	---	1.50 (0.52, 4.35)
Education		
No Education	Reference	
Primary	1.15 (0.46, 2.87)	---
Junior or Senior	0.93 (0.37, 2.32)	---
University	0.70 (0.24, 2.03)	---
Marital Status		
Never Married	Reference	Reference
Married	2.35 (0.96, 5.74)	1.46 (0.53, 4.02)
Other	0.51 (0.15, 1.74)	5.01 (0.80, 31.17)
Employment		
Not Working		Reference
Agriculture-based Labor	0.60 (0.32, 1.11)	1.66 (0.49, 5.70)
Skilled Manual Labor^c^	0.45 (0.21, 0.93) *	0.69 (0.19, 2.47)
Skilled Labor^d^	0.97 (0.64, 1.49)	1.28 (0.38, 4.26)
Overweight (BMI ≥ 25 kg/m^2^)		
No	Reference	Reference
Yes	2.42 (1.67, 3.51) *	2.26 (1.19, 4.30) *
Physical Activity (Last Week)^e^:		
No Vigorous Activity	Reference	Reference
Vigorous Activity	0.66 (0.37, 1.18)	0.63 (0.35, 1.12)
Consumed (Last Week):		
Fast Food		
No	Reference	
Yes	1.04 (0.59, 1.83)	---
Mean Number of Days Consumed Last Week^f^:		
Instant Noodles	1.12 (1.01, 1.25) *	---
Soda	---	1.32 (1.10, 1.58) *
Household Level		
Residence		
Rural	Reference	Reference
Urban	1.51 (1.00, 2.31) *	1.49 (0.78, 2.86)
Wealth		
Lowest	Reference	Reference
Second	1.05 (0.60, 1.84)	1.34 (0.53, 3.43)
Middle	0.84 (0.47, 1.51)	1.24 (0.44, 3.46)
Fourth	0.90 (0.52, 1.56)	0.47 (0.18, 1.25)
Highest	0.75 (0.43, 1.32)	1.22 (0.51, 2.91)

BMI = body mass index, CI = confidence interval; hs-CRP = high sensitivity c-reactive protein

^a^ Defined as hs-CRP ≥ 3

^b^ Odds ratios and confidence intervals are estimated using logistic regression and are weighted to account for the survey design. Models exclude women who are currently pregnant.

^c^ Skilled manual labor combines the following employment sectors: mining, manufacturing, electric, gas, water maintenance, and construction

^d^ Skilled labor combines the following employment sectors: retail and service, transportation

^e^ Defined using the International Physical Activity Questionnaire

^f^ Modelled as a continuous variable, the average number of days consumed is queried if the respondent reported that they consumed in the last week (i.e. these models exclude non-consumers).

* *p* < 0.05

Among men, being overweight was associated with more than 2-fold higher odds of elevated hs-CRP (OR = 2.26; 95% CI: 1.19, 4.30). Every additional day men consumed soda was associated with 32% higher odds of elevated hs-CRP (OR = 1.32; 95% CI: 1.10, 1.58).

### Central obesity

Older women and those with higher educational attainment had higher odds of central obesity (Table 4). Unexpectedly, moderate physical activity (versus no activity) was associated with higher odds of central obesity, among women (OR = 1.18; 95% CI: 1.01, 1.39). Higher household expenditures on cooking oil (versus lower expenditures) (OR = 1.36; 95% CI: 1.16, 1.60) and urban residence (OR = 1.49; 95% CI: 1.26, 1.77) also predicted higher odds of central obesity. But working in agriculture-based employment (versus unemployment) was associated with lower odds of central obesity (OR = 0.60; 95% CI: 0.48, 0.75), as was smoking (versus non-smoking) (OR = 0.62; 95% CI: 0.43, 0.91).

**Table 4:** Multivariable Logistic Regression Testing the association between selected characteristics and central obesity among adults in Indonesia, 2014.

	Women^a^^,^^b^	Men^a^^,^^b^
	N = 4,061	N = 4,523
Individual Level	Odds Ratio (95% CI)	Odds Ratio (95% CI)
Age (in years)		
40–49	Reference	Reference
50–59	1.23 (1.02, 1.47) *	1.26 (1.04, 1.52) *
≥ 60	1.01 (0.80, 1.27)	0.88 (0.69, 1.12)
Education		
No Education	Reference	Reference
Primary	1.65 (1.28, 2.12) *	1.63 (0.97, 2.76)
Junior or Senior	1.88 (1.40, 2.53) *	2.42 (1.41, 4.15) *
University	1.81 (1.22, 2.68) *	3.27 (1.84, 5.82) *
Marital Status		
Never Married	Reference	
Married	1.46 (0.54, 3.95)	---
Other	1.25 (0.45, 3.49)	---
Employment		
Not Working	Reference	Reference
Agriculture-based Labor	0.60 (0.48, 0.75) *	0.42 (0.31, 0.58) *
Skilled Manual Labor^c^	0.88 (0.64, 1.22)	0.55 (0.39, 0.76) *
Skilled Labor^d^	1.11 (0.91, 1.35)	1.01 (0.75, 1.35)
Smoking Status		
Does not smoke	Reference	Reference
Currently Smoking	0.62 (0.43, 0.91) *	0.60 (0.51, 0.71) *
Physical Activity (Last Week)^e^:		
No Vigorous Activity	Reference	Reference
Vigorous Activity	0.81 (0.63, 1.04)	0.78 (0.64, 0.94) *
No Moderate Activity	Reference	
Moderate Activity	1.18 (1.01, 1.39) *	---
No Walking	Reference	
Walking	0.91 (0.76, 1.09)	---
Consumed (Last Week):		
Fast Food		
No	Reference	Reference
Yes	1.07 (0.77, 1.49)	1.28 (0.91, 1.80)
Soda		
No		Reference
Yes	---	1.32 (1.07, 1.64) *
Fried Snacks		
No	Reference	Reference
Yes	1.03 (0.88, 1.22)	1.17 (0.98, 1.39)
Household Level		
Food Expenditures^f^		
Cooking oil Lowest	Reference	Reference
Cooking oil Highest	1.36 (1.16, 1.60) *	1.12 (0.95, 1.32)
Residence		
Rural	Reference	Reference
Urban	1.49 (1.26, 1.77) *	1.28 (1.06, 1.55) *
Wealth		
Lowest	Reference	Reference
Second	1.02 (0.81, 1.28)	1.08 (0.85, 1.37)
Middle	1.18 (0.92, 1.52)	1.02 (0.78, 1.34)
Fourth	1.00 (0.78, 1.27)	0.95 (0.74, 1.23)
Highest	1.11 (0.88, 1.42)	1.14 (0.88, 1.47)

CI = confidence interval

^a^ Measured among adults ≥ 40 years old. Defined as waist circumference ≥ 90 cm if male and waist circumference ≥ 80 cm if female

^b^ Odds ratios and confidence intervals are estimated using logistic regression and are weighted to account for the survey design. Models exclude women who are currently pregnant.

^c^ Skilled manual labor combines the following employment sectors: mining, manufacturing, electric, gas, water maintenance, and construction

^d^ Skilled labor combines the following employment sectors: retail and service, transportation

^e^ Defined using the International Physical Activity Questionnaire

^f^ Indicates the household level expenditure on each item as a percentage of the households’ total expenditures on food

* *p* < 0.05

Similarly, among men, older age (OR = 1.26; 95% CI: 1.04, 1.52), higher educational attainment (OR = 3.27; 95% CI: 1.84, 5.82) and urban residence (OR = 1.28; 95% CI: 1.06, 1.55) predicted higher central obesity, as did soda consumption (OR = 1.32; 95% CI: 1.07, 1.64). On the contrary, smoking (OR = 0.60; 95% CI: 0.51, 0.71) and vigorous physical activity (OR = 0.78; 95% CI: 0.64, 0.94) were associated with lower odds of central obesity, as was working in agriculture-based employment (OR = 0.42; 95% CI: 0.31, 0.58) and skilled manual labor (OR = 0.55; 95% CI: 0.39, 0.76).

### Type 2 diabetes

Among women, being aged ≥ 40 years (versus 19–29 years old) was associated with a 3- to 8-times higher odds of diabetes (Table 5). Being overweight was associated with 2.5-fold higher odds of diabetes (OR = 2.54; 95% CI: 1.73, 3.75).

**Table 5:** Multivariable Logistic Regression Testing the association between selected characteristics and Type 2 diabetes among adults in Indonesia, 2014.

	Women^a^^,^^b^	Men^a^^,^^b^
	N = 2,045	N = 1,596
Individual Level	Odds Ratio (95% CI)	Odds Ratio (95% CI)
Age (in years)		
19–29	Reference	Reference
30–39	2.90 (0.96, 8.70)	5.45 (0.89, 33.50)
40–49	4.53 (1.56, 13.19) *	6.88 (0.92, 51.39)
50–59	7.50 (2.69, 20.93) *	10.66 (1.53, 74.33) *
≥ 60	8.09 (2.86, 22.87) *	14.59 (4.65, 45.80) *
Education		
No Education		Reference
Primary	---	4.89 (1.07, 22.31) *
Junior or Senior	---	5.71 (1.18, 27.66) *
University	---	6.66 (1.24, 35.74) *
Marital Status		
Never Married		
Married	---	0.50 (0.09, 2.73)
Other	---	0.43 (0.05, 3.64)
Employment		
Not Working		Reference
Agriculture-based Labor	---	0.56 (0.28, 1.12)
Skilled Manual Labor^c^	---	0.59 (0.27, 1.27)
Skilled Labor^d^	---	0.89 (0.48, 1.63)
Overweight (BMI ≥ 25 kg/m^2^)		
No	Reference	Reference
Yes	2.54 (1.73, 3.75) *	5.10 (3.21, 8.10) *
Smoking Status		
Does not smoke		Reference
Currently Smoking	---	1.18 (0.76, 1.82)
Physical Activity (Last Week)^e^:		
No Vigorous Activity		Reference
Vigorous Activity	---	0.60 (0.35, 1.05)
No Moderate Activity		Reference
Moderate Activity	---	0.72 (0.47, 1.11)
Consumed (Last Week):		
Instant Noodles		
No	Reference	
Yes	0.92 (0.64, 1.32)	---
Soda		
No	Reference	
Yes	0.62 (0.32, 1.21)	---
Household Level		
Residence		
Rural	Reference	Reference
Urban	1.30 (0.91, 1.87)	0.98 (0.61, 1.56)
Wealth		
Lowest	Reference	Reference
Second	1.09 (0.66, 1.82)	1.77 (0.96, 3.28)
Middle	1.69 (0.97, 2.96)	1.09 (0.54, 2.19)
Fourth	1.34 (0.78, 2.27)	1.46 (0.74, 2.91)
Highest	1.20 (0.69, 2.08)	1.32 (0.63, 2.78)

BMI = body mass index, CI = confidence interval

^a^ Defined as HbA_1c_ ≥ 6.5%

^b^ Odds ratios and confidence intervals are estimated using logistic regression and are weighted to account for the survey design. Models exclude women who are currently pregnant.

^c^ Skilled manual labor combines the following employment sectors: mining, manufacturing, electric, gas, water maintenance, and construction

^d^ Skilled labor combines the following employment sectors: retail and service, transportation

^e^ Defined using the International Physical Activity Questionnaire

* *p* < 0.05

Compared to men aged 19–29 years, being ≥ 30 years old was associated with 5- to 15-fold higher odds of diabetes. Being overweight was associated with 5-fold higher odds of diabetes (OR = 5.10; 95% CI: 3.21, 8.10). Compared to men with no education, having a primary (OR = 4.89; 95% CI: 1.07, 22.31), junior/senior (OR = 5.71; 95%: 1.18, 27.66) or university education (OR = 6.66; 95%: 1.24, 35.74) was also associated with higher odds of diabetes.

Overall, results were similar in magnitude, direction, and statistical significance when overweight was defined as BMI ≥ 23 kg/m^2^. Unlike our main findings, when defining overweight as BMI ≥ 23 kg/m^2^, higher wealth was associated with lower odds of hypertension, among men (OR = 0.78; 95% CI: 0.61, 0.99) (S5 Table). In sensitivity analyses, overweight (defined as BMI ≥ 23 kg/m^2^) was not associated with elevated hs-CRP, among men (OR = 1.63; 95% CI: 0.87, 3.05) (S6 Table).

## Discussion

Large segments of the adult population in Indonesia now have or are at risk for NCDs. Key metabolic determinants in the progression to CVD, including hypertension, elevated hs-CRP, and central obesity, as well as type 2 diabetes, were prevalent. About 20% of adults were hypertensive in 2014. At the same time, one-fifth of the adult population had elevated hs-CRP. Of particular concern were the very high prevalence of central obesity among women (~70%) and, compared to global estimates, a relatively high prevalence of diabetes among women: 11.5%. Older-age and being overweight were consistently associated with chronic disease-related health. Urban area residence (versus rural) was associated with elevated hs-CRP (women) and central obesity (women and men). Consuming instant noodles (women) and soda (men) were associated with elevated hs-CRP and soda consumption was associated with higher central obesity among men. On the contrary, vigorous physical activity was associated with lower odds of central obesity among men.

Higher odds of hypertension were observed among older and overweight adults in this sample. Higher education was associated with lower odds of hypertension among women, as was being employed (versus non-employed) among both women and men. These findings are generally consistent with prior studies conducted in Indonesia [46–51] and Asia [52], which suggest social determinants play a role in the development of hypertension.

Being overweight, living in urban area, and an unhealthful diet were associated with higher odds of elevated hs-CRP, in this sample. These factors are all inter-related; urban-area residence results in an increased consumption of unhealthy foods and decreased physical activity, which are associated with higher overweight [36,53–55]. In turn, being overweight is a well-established risk factor for elevated hs-CRP [56]. For example, in a seminal U.S.-based study, obese men were 2-times as likely and obese women 6-times as likely to have elevated CRP levels [56]. Relatively few studies have explored the determinants of elevated hs-CRP in lower-income countries, but a strong association between obesity and hs-CRP has also been reported among adults in Jamaica [57] and among Filipinos [58]. Both hypertension and elevated hs-CRP were associated with CVD [59,60] and elevated hs-CRP is associated with higher diabetes risk [61].

Similar to prior studies in Asia [62–64], the prevalence of central obesity was very high among women (~70%), compared to only 24% among men. In multivariable models, age, education, and urban residence were associated with higher odds of central obesity, while agriculture-based employment and smoking were associated with lower odds of central obesity. One other Indonesian study also suggested that physical labor was related to lower central obesity among older-aged adults [63] and agriculture-based employment was associated with lower odds of overweight among women across 40 lower-income countries [65]. Central obesity is associated with higher mortality risk [66] and higher CVD among Asians [67]. Notably, Asians tend to develop CVD and diabetes with a lower BMI than individuals in other regions, due in part to their propensity to store adipose tissue around their waist [45,68]. Therefore, it is especially important to consider the prevalence of central obesity in this population, which could suggest that the risk for NCDs among women is more widespread than previously thought [69,70].

Findings from these analyses also indicate larger portions of women (versus men) have diabetes; this is similar to the 2013 National Health Survey, which found 10.1% of women and 7.3% of men were diabetic [16,71]. Prevalence estimates from this sample are higher than estimates from the NCD Risk Factor Collaboration study for women, which reported that the global prevalence of diabetes was 7.9% [72]. High diabetes prevalence in Indonesia suggests that there may be Southeast Asia-specific risk factors to consider. In addition to the aforementioned risk of diabetes with lower BMIs, physiological responses to food could vary between populations. For example, the glucose response in Asians is higher than Caucasians in the U.S. [73]. In this sample, older age, being overweight, and higher education (among men) were associated with higher odds of diabetes. This is consistent with prior studies that show that age induces a decrease of insulin sensitivity and/or insulin resistance [74] and that age is associated with a higher odds of diabetes prevalence, using regression-based methods [52,75]. This is also consistent with 2007 data, which found that age and obesity were associated with diabetes in Indonesia [76]. In multivariable models, higher education has also previously been associated with diabetes in Asia [52].

Consuming instant noodles and soda were associated with elevated hs-CRP and central obesity. Some associations between an unhealthy diet and hs-CRP and central obesity, as well as the consistent associations between overweight and hypertension, elevated hs-CRP, and type 2 diabetes in these analyses, suggests that the nutrition transition has played a major role in these trends. There have been marked changes in Indonesia’s food system and a reduction in energy expenditure. More calories are now available, with an estimated 20% coming from fat [70], in part due to the ubiquitous use of vegetable-based cooking oils, that are high in trans- and saturated fats. Higher caloric intake has been associated with weight gain and the consumption of trans fats, which has been associated with increased cardiometabolic risk (51) and insulin resistance (52). Individual-level data from this sample echo trends from macro-level data, as about 60% of adults in this sample reported regularly consuming fried snacks. In addition, polished rice and refined wheat, which are high glycemic index foods, form the basis of many Indonesians’ diets; consuming high glycemic index foods is associated with a 2-fold increased risk of diabetes [77]. In addition, by one account, Indonesians are consuming 15 grams of salt per day, which is about 3-times the recommended amount [78], and strongly related to hypertension risk [24,25].

Although there were not consistent associations between physical activity and NCD-related outcomes, vigorous physical activity was associated with lower odds of central obesity among men. Some prior studies from Indonesia have also reported no associations between physical activity and hypertension [50] and between activity and central obesity, in samples that pool men and women [79]. Contrary to findings reported in these analyses, results from a robust meta-analysis, which largely includes data from high-income countries, reports that physical activity is strongly related to hypertension [80]. Physical activity has also been associated with insulin sensitivity in Indonesia [76] and reduced incidence of diabetes [81–83] in prior studies. Overall, the literature and findings from this study suggest that interventions that promote healthful eating and physical activity, in order to reduce the prevalence of NCDs, warrant consideration in Indonesia.

Notably, very few respondents reported taking medication for hypertension or diabetes. This could suggest that these adults are undiagnosed and/or do not have regular access to health care [84]. One prior study suggests that approximately 70% of all diabetes cases in Indonesia remain undiagnosed [85]. The Government has undertaken several WHO-recommended initiatives designed to strengthen the primary healthcare system, including implementing coverage and service delivery reforms under *Jaminan Kesehatan Nasional*, the national health insurance plan [86]. But current evidence suggests that prevention and management of diet-related NCDs remain a challenge [21,86–90].

Study limitations warrant consideration. Due to our study design, we cannot establish temporality or rule out unmeasured or residual confounding. Diet and physical activity data are self-reported and only capture a limited picture of the consumption of ultra-processed foods and physical activities. Results for hs-CRP and HbA_1C_ are estimated using DBS, therefore, these results may not be comparable to those that that involve assay results from venous blood. In addition, we examine the risk factors for a single outcome in separate models, and this does not provide information on risk factors for co-morbidities (e.g. co-existing hypertension and diabetes).

### Conclusions

We find a relatively high prevalence of nutrition-related chronic diseases among Indonesians. Moreover, overweight was consistently associated with hypertension, hs-CRP, and diabetes. These findings provide preliminary empirical evidence that interventions that target healthful food intake (e.g. reduce the intake of ultra-processed foods) and physical activity should be considered and that the reduction of overweight is critical for preventing chronic diseases in Indonesia. Future studies should investigate risk factors for co-morbidities.

## Supporting information

S1 Table Univariate Logistic Regression Testing the association between selected characteristics and hypertension among adults in Indonesia, 2014. (DOCX)

S2 Table Univariate Logistic Regression Testing the association between selected characteristics and elevated hs-CRP among adults in Indonesia, 2014. (DOCX)

S3 Table Univariate Logistic Regression Testing the association between selected characteristics and central obesity among adults in Indonesia, 2014. (DOCX)

S4 Table Univariate Logistic Regression Testing the association between selected characteristics and Type 2 diabetes among adults in Indonesia, 2014. (DOCX)

S5 Table Multivariable Logistic Regression Testing the association between selected characteristics and hypertension among adults in Indonesia, 2014. (DOCX)

S6 Table Multivariable Logistic Regression Testing the association between selected characteristics and elevated hs-CRP among adults in Indonesia, 2014. (DOCX)

S7 Table Multivariable Logistic Regression Testing the association between selected characteristics and Type 2 diabetes among adults in Indonesia, 2014. (DOCX)
