# Supplementary material for: Risk factors for nutrition-related chronic disease among adults in Indonesia
Source: PLoS One. 2019 Aug 30;14(8):e0221927. doi: 10.1371/journal.pone.0221927 (PMC6716634; doi:10.1371/journal.pone.0221927)
Supplement: S1 Table — (DOCX) [file pone.0221927.s001.docx]

**S1 Table. Univariate Logistic Regression Testing the Association Between Selected Characteristics and Hypertension Among Adults in Indonesia, 2014**

|  | **Women^a,b^** | | **Men^a,b^** | | |
| --- | --- | --- | --- | --- | --- |
|  | n | Odds Ratio  (95% CI) | | n | Odds Ratio  (95% CI) |
| Individual Level |  |  | |  |  |
| Age (in years) |  |  | |  |  |
| 19-29 | 4,340 | Reference | | 3,389 | Reference |
| 30-39 | 4,443 | 2.80 (2.36, 3.32) * | | 3,982 | 1.35 (1.16, 1.58) * |
| 40-49 | 2,816 | 7.63 (6.44, 9.03) * | | 2,766 | 2.95 (2.53, 3.43) * |
| 50-59 | 2,061 | 15.49 (13.02, 18.42) * | | 1,751 | 5.36 (4.56, 6.29) * |
| ≥ 60 | 1,919 | 32.02 (26.71, 38.38) * | | 1,644 | 10.71 (9.10, 12.60) * |
|  |  |  | |  |  |
| Education |  |  | |  |  |
| No Education | 1,070 | Reference | | 387 | Reference |
| Primary | 4,998 | 0.65 (0.56, 0.77) * | | 4,192 | 0.67 (0.52, 0.86) * |
| Junior or Senior | 4,939 | 0.27 (0.23, 0.32) * | | 4,722 | 0.40 (0.31, 0.51) * |
| University | 1,612 | 0.21 (0.17, 0.26) * | | 1,465 | 0.56 (0.42, 0.73) * |
|  |  |  | |  |  |
| Marital Status |  |  | |  |  |
| Never Married | 1,282 | Reference | | 2,168 | Reference |
| Married | 12,062 | 3.54 (2.76, 4.54) * | | 10,782 | 2.46 (2.14, 2.83) * |
| Other | 2,231 | 10.79 (8.30, 14.03) * | | 577 | 5.08 (4.03, 6.39) * |
|  |  |  | |  |  |
| Employment |  |  | |  |  |
| Not Working | 5,839 | Reference | | 1,190 | Reference |
| Agriculture-based Labor | 2,392 | 0.87 (0.77, 0.98) * | | 3,530 | 0.53 (0.45, 0.62) * |
| Skilled Manual Labor^c^ | 1,332 | 0.60 (0.51, 0.71) * | | 2,843 | 0.34 (0.29, 0.40) * |
| Skilled Labor^d^ | 5,747 | 0.62 (0.57, 0.69) * | | 5,731 | 0.44 (0.38, 0.51) * |
|  |  |  | |  |  |
| Overweight/Obese |  |  | |  |  |
| No | 8,778 | Reference | | 10,229 | Reference |
| Yes | 6,102 | 1.63 (1.49, 1.77) * | | 3,240 | 2.35 (2.12, 2.59) * |
|  |  |  | |  |  |
| Smoking Status |  |  | |  |  |
| Does not smoke | 15,055 | Reference | | 4,407 | Reference |
| Currently Smoking | 442 | 1.73 (1.39, 2.17) * | | 9,056 | 0.64 (0.58, 0.70) * |
|  |  |  | |  |  |
| Physical Activity in the Last Week^e^: |  |  | |  |  |
| No Vigorous Activity | 13,235 | Reference | | 8,179 | Reference |
| Vigorous Activity | 1,552 | 0.94 (0.82, 1.09) | | 4,658 | 0.68 (0.61, 0.75) * |
|  |  |  | |  |  |
| No Moderate Activity | 6,121 | Reference | | 6,004 | Reference |
| Moderate Activity | 8,666 | 0.78 (0.71, 0.85) * | | 6,833 | 0.78 (0.71, 0.86) * |
|  |  |  | |  |  |
| No Walking | 4,772 | Reference | | 3,558 | Reference |
| Walking | 10,015 | 1.03 (0.94, 1.13) | | 9,279 | 1.14 (1.03, 1.27) * |
|  |  |  | |  |  |
| Consumed in the Last Week: |  |  | |  |  |
| *Instant Noodles* |  |  | |  |  |
| No | 5,248 | Reference | | 4,365 | Reference |
| Yes | 9,532 | 0.62 (0.56, 0.67) * | | 8,463 | 0.65 (0.59, 0.71) * |
|  |  |  | |  |  |
| *Fast Food* |  |  | |  |  |
| No | 13,142 | Reference | | 11,586 | Reference |
| Yes | 1,638 | 0.64 (0.55, 0.75) * | | 1,242 | 0.76 (0.64, 0.90) * |
|  |  |  | |  |  |
| *Soda* |  |  | |  |  |
| No | 12,927 | Reference | | 9,683 | Reference |
| Yes | 1,853 | 0.69 (0.60, 0.80) * | | 3,145 | 0.73 (0.65, 0.82) * |
|  |  |  | |  |  |
| *Fried Snacks* |  |  | |  |  |
| No | 5,347 | Reference | | 4,367 | Reference |
| Yes | 9,433 | 0.99 (0.90, 1.08) | | 8,461 | 0.93 (0.84, 1.03) |
|  |  |  | |  |  |
| Mean Number of Days Consumed in the Last Week^f^: |  |  | |  |  |
| Instant Noodles | 9,532 | 0.95 (0.92, 0.99) * | | 8,463 | 0.96 (0.93, 1.00) |
| Fast Food | 1,638 | 0.95 (0.84, 1.06) | | 1,242 | 1.01 (0.87, 1.17) |
| Soda | 1,853 | 0.95 (0.86, 1.05) | | 3,145 | 0.96 (0.89, 1.03) |
| Fried Snacks | 9,433 | 1.02 (0.99, 1.04) | | 8,461 | 1.02 (0.99, 1.05) |
| Household Level |  |  | |  |  |
| Food Expenditures^g^ |  |  | |  |  |
| *Rice* |  |  | |  |  |
| Lowest | 8,145 | Reference | | 7,044 | Reference |
| Highest | 7,412 | 0.92 (0.84, 1.00) | | 6,449 | 0.90 (0.82, 0.99) * |
|  |  |  | |  |  |
| *Cooking oil* |  |  | |  |  |
| Lowest | 9,363 | Reference | | 8,236 | Reference |
| Highest | 6,192 | 0.92 (0.84, 1.00) * | | 5,255 | 1.06 (0.96, 1.16) |
|  |  |  | |  |  |
| Residence |  |  | |  |  |
| Rural | 6,406 | Reference | | 5,586 | Reference |
| Urban | 9,173 | 1.02 (0.94, 1.11) | | 7,944 | 1.04 (0.95, 1.14) |
|  |  |  | |  |  |
| Wealth |  |  | |  |  |
| Lowest | 2,895 | Reference | | 2,852 | Reference |
| Second | 2,611 | 0.90 (0.78, 1.04) | | 2,491 | 0.83 (0.71, 0.96) * |
| Middle | 1,880 | 0.90 (0.76, 1.05) | | 1,823 | 0.95 (0.81, 1.11) |
| Fourth | 2,183 | 0.79 (0.68, 0.91) * | | 2,074 | 0.92 (0.79, 1.07) |
| Highest | 2,051 | 0.83 (0.71, 0.96) * | | 1,927 | 0.90 (0.77, 1.05) |
|  |  |  | |  |  |
| Family Size |  |  | |  |  |
| ≤ 4 | 9,804 | Reference | | 9,804 | Reference |
| > 4 | 5,775 | 0.87 (0.80, 0.95) * | | 5,775 | 0.90 (0.82, 1.00) |

CI = confidence interval

^a^ Systolic and diastolic blood pressure are based on the average of three measurements. Hypertension is defined as systolic blood pressure ≥140 or diastolic blood pressure ≥ 90 and/or current use of antihypertensive medication

^b^ Odds ratios and confidence intervals are estimated using logistic regression and are weighted to account for the survey design. Models exclude women who are currently pregnant.

^c^ Skilled manual labor combines the following employment sectors: mining, manufacturing, electric, gas, water maintenance, and construction

^d^ Skilled labor combines the following employment sectors: retail and service, transportation

^e^ Defined using the International Physical Activity Questionnaire

^f^ Modeled as a continuous variable, the average number of days consumed is queried if the respondent reported that they consumed in the last week

^g^ Indicates the household level expenditure on each item as a percentage of the households’ total expenditures on food

* *p* < 0.05
