# Supplementary material for: Risk factors for nutrition-related chronic disease among adults in Indonesia
Source: PLoS One. 2019 Aug 30;14(8):e0221927. doi: 10.1371/journal.pone.0221927 (PMC6716634; doi:10.1371/journal.pone.0221927)
Supplement: S2 Table — (DOCX) [file pone.0221927.s002.docx]

**S2 Table. Univariate Logistic Regression Testing the Association Between Selected Characteristics and Elevated hs-CRP Among Adults in Indonesia, 2014**

|  | **Women^a,b^** | | **Men^a,b^** | |
| --- | --- | --- | --- | --- |
|  | n | Odds Ratio  (95% CI) | n | Odds Ratio  (95% CI) |
| Individual Level |  |  |  |  |
| Age (in years) |  |  |  |  |
| 19-29 | 736 | Reference | 693 | Reference |
| 30-39 | 765 | 1.12 (0.85, 1.47) | 648 | 1.30 (0.91, 1.87) |
| 40-49 | 455 | 1.10 (0.80, 1.50) | 410 | 0.92 (0.60, 1.40) |
| 50-59 | 608 | 1.14 (0.86, 1.52) | 330 | 1.46 (0.95, 2.24) |
| ≥ 60 | 872 | 0.89 (0.68, 1.17) | 711 | 1.50 (1.07, 2.12) * |
|  |  |  |  |  |
| Education |  |  |  |  |
| No Education | 372 | Reference | 106 | Reference |
| Primary | 1,221 | 1.40 (0.99, 1.98) | 911 | 0.85 (0.45, 1.58) |
| Junior or Senior | 881 | 1.76 (1.23, 2.52) * | 861 | 0.78 (0.42, 1.45) |
| University | 289 | 1.70 (1.08, 2.66) * | 272 | 1.02 (0.51, 2.04) |
|  |  |  |  |  |
| Marital Status |  |  |  |  |
| Never Married | 309 | Reference | 574 | Reference |
| Married | 2,379 | 1.54 (1.03, 2.30) * | 2,055 | 1.40 (1.01, 1.96) * |
| Other | 746 | 1.16 (0.74, 1.80) | 163 | 1.80 (1.05, 3.10) * |
|  |  |  |  |  |
| Employment |  |  |  |  |
| Not Working | 1,311 | Reference | 395 | Reference |
| Agriculture-based Labor | 574 | 0.56 (0.41, 0.77) * | 793 | 0.64 (0.43, 0.95) * |
| Skilled Manual Labor^c^ | 283 | 0.68 (0.48, 0.99) * | 509 | 0.60 (0.40, 0.91) * |
| Skilled Labor^d^ | 1,193 | 1.03 (0.82, 1.28) | 1,045 | 0.83 (0.58, 1.20) |
|  |  |  |  |  |
| Overweight/Obese |  |  |  |  |
| No | 2,004 | Reference | 2,179 | Reference |
| Yes | 1,304 | 2.54 (2.07, 3.11) * | 595 | 1.87 (1.42, 2.47) * |
|  |  |  |  |  |
| Smoking Status |  |  |  |  |
| Does not smoke | 3,266 | Reference | 974 | Reference |
| Currently Smoking | 143 | 0.74 (0.46, 1.19) | 1,802 | 0.80 (0.62, 1.04) |
|  |  |  |  |  |
|  |  |  |  |  |
| Physical Activity in the Last Week^e^: |  |  |  |  |
|  |  |  |  |  |
| No Vigorous Physical Activity | 2,801 | Reference | 1,640 | Reference |
| Vigorous Physical Activity | 350 | 0.60 (0.43, 0.84) * | 960 | 0.75 (0.56, 0.99) * |
|  |  |  |  |  |
| No Moderate Physical Activity | 1,393 | Reference | 1,228 | Reference |
| Moderate Physical Activity | 1,758 | 1.07 (0.87, 1.31) | 1,372 | 0.95 (0.73, 1.23) |
|  |  |  |  |  |
| No Walking | 988 | Reference | 699 | Reference |
| Any Walking | 2,163 | 0.94 (0.76, 1.17) | 1,901 | 0.90 (0.67, 1.20) |
|  |  |  |  |  |
| Consumed in the Last Week: |  |  |  |  |
| *Instant Noodles* |  |  |  |  |
| No | 1,308 | Reference | 955 | Reference |
| Yes | 1,842 | 1.07 (0.87, 1.31) | 1,643 | 0.88 (0.67, 1.15) |
|  |  |  |  |  |
| *Fast Food* |  |  |  |  |
| No | 2,846 | Reference | 2,341 | Reference |
| Yes | 304 | 1.43 (1.03, 1.97) * | 257 | 0.90 (0.60, 1.35) |
|  |  |  |  |  |
| *Soda* |  |  |  |  |
| No | 2,808 | Reference | 1,979 | Reference |
| Yes | 342 | 1.24 (0.90, 1.70) | 619 | 1.11 (0.83, 1.49) |
|  |  |  |  |  |
| *Fried Snacks* |  |  |  |  |
| No | 1,194 | Reference | 917 | Reference |
| Yes | 1,956 | 1.10 (0.90, 1.35) | 1,681 | 1.10 (0.84, 1.46) |
|  |  |  |  |  |
| Mean Number of Days Consumed in the Last Week^f^: |  |  |  |  |
| Instant Noodles | 1,842 | 1.10 (1.02, 1.19) * | 1,643 | 1.07 (0.98, 1.17) |
| Fast Food | 304 | 0.91 (0.74, 1.13) | 257 | 0.82 (0.55, 1.21) |
| Soda | 342 | 1.00 (0.83, 1.22) | 619 | 1.22 (1.05, 1.42) * |
| Fried Snacks | 1,956 | 1.04 (0.98, 1.10) | 1,681 | 1.02 (0.96, 1.10) |
| Household Level |  |  |  |  |
| Food Expenditures^g^ |  |  |  |  |
| *Rice* |  |  |  |  |
| Lowest | 1,913 | Reference | 1,525 | Reference |
| Highest | 1,515 | 1.09 (0.90, 1.33) | 1,258 | 0.92 (0.71, 1.19) |
|  |  |  |  |  |
| *Cooking oil* |  |  |  |  |
| Lowest | 2,104 | Reference | 1,717 | Reference |
| Highest | 1,325 | 0.87 (0.71, 1.07) | 1,066 | 1.02 (0.79, 1.31) |
|  |  |  |  |  |
| Residence |  |  |  |  |
| Rural | 1,443 | Reference | 1,167 | Reference |
| Urban | 1,993 | 1.52 (1.25, 1.86) * | 1,624 | 1.20 (0.93, 1.54) |
|  |  |  |  |  |
| Wealth |  |  |  |  |
| Lowest | 591 | Reference | 576 | Reference |
| Second | 546 | 0.85 (0.60, 1.19) | 500 | 0.98 (0.66, 1.46) |
| Middle | 403 | 0.80 (0.56, 1.16) | 326 | 0.99 (0.63, 1.56) |
| Fourth | 456 | 0.83 (0.59, 1.17) | 429 | 0.75 (0.49, 1.14) |
| Highest | 456 | 0.71 (0.50, 1.00) | 382 | 0.95 (0.62, 1.47) |
|  |  |  |  |  |
| Family Size |  |  |  |  |
| ≤ 4 | 2,134 | Reference | 1,719 | Reference |
| > 4 | 1,302 | 0.90 (0.74, 1.11) | 1,072 | 1.17 (0.91, 1.51) |

CI = confidence interval; hs-CRP = high sensitivity c-reactive protein

^a^ Defined as hs-CRP > 3 mg/dL

^b^ Odds ratios and confidence intervals are estimated using logistic regression and are weighted to account for the survey design. Models exclude women who are currently pregnant.

^c^ Skilled manual labor combines the following employment sectors: mining, manufacturing, electric, gas, water maintenance, and construction

^d^ Skilled labor combines the following employment sectors: retail and service, transportation

^e^ Defined using the International Physical Activity Questionnaire

^f^ Modeled as a continuous variable, the average number of days consumed is queried if the respondent reported that they consumed item in the last week

^g^ Indicates the household level expenditure on each item as a percentage of the households’ total expenditures on food

* *p* < 0.05
