# Supplementary material for: Risk factors for nutrition-related chronic disease among adults in Indonesia
Source: PLoS One. 2019 Aug 30;14(8):e0221927. doi: 10.1371/journal.pone.0221927 (PMC6716634; doi:10.1371/journal.pone.0221927)
Supplement: S3 Table — (DOCX) [file pone.0221927.s003.docx]

**S3 Table. Univariate Logistic Regression Testing the Association Between Selected Characteristics and Central Obesity Among Adults in Indonesia, 2014**

|  | **Women^a,b^** | | **Men^a,b^** | |
| --- | --- | --- | --- | --- |
|  | n | Odds Ratio  (95% CI) | n | Odds Ratio  (95% CI) |
| Individual Level |  |  |  |  |
| Age (in years) |  |  |  |  |
| 40-49 | 2,816 | Reference | 2,771 | Reference |
| 50-59 | 2,061 | 1.11 (0.96, 1.27) | 1,751 | 1.05 (0.90, 1.22) |
| ≥ 60 | 1,880 | 0.74 (0.64, 0.85) * | 1,614 | 0.66 (0.56, 0.78) * |
|  |  |  |  |  |
| Education |  |  |  |  |
| No Education | 968 | Reference | 344 | Reference |
| Primary | 3,223 | 2.24 (1.90, 2.66) * | 2,699 | 2.67 (1.68, 4.23) * |
| Junior or Senior | 1,490 | 3.28 (2.70, 3.99) * | 1,838 | 5.87 (3.70, 9.32) * |
| University | 465 | 4.12 (3.11, 5.47) * | 630 | 11.01 (6.80, 17.84) * |
| Marital Status |  |  |  |  |
| Never Married | 131 | Reference | 112 | Reference |
| Married | 4,708 | 2.23 (1.49, 3.33) * | 5,608 | 1.43 (0.82, 2.48) |
| Other | 1,915 | 1.59 (1.06, 2.40) * | 416 | 0.83 (0.45, 1.54) |
|  |  |  |  |  |
| Employment |  |  |  |  |
| Not Working | 2,344 | Reference | 672 | Reference |
| Agriculture-based Labor | 1,408 | 0.55 (0.47, 0.64) * | 2,006 | 0.46 (0.36, 0.58) * |
| Skilled Manual Labor^c^ | 458 | 0.86 (0.68, 1.08) | 1,020 | 0.74 (0.57, 0.95) * |
| Skilled Labor^d^ | 2,417 | 1.42 (1.23, 1.63) * | 2,328 | 1.68 (1.36, 2.08) * |
|  |  |  |  |  |
| Smoking Status |  |  |  |  |
| Does not smoke | 6,363 | Reference | 2,200 | Reference |
| Currently Smoking | 344 | 0.52 (0.41, 0.67) * | 3,904 | 0.48 (0.42, 0.55) * |
|  |  |  |  |  |
| Physical Activity in the Last Week^e^: |  |  |  |  |
| No Vigorous Activity | 5,484 | Reference | 3,901 | Reference |
| Vigorous Activity | 689 | 0.75 (0.63, 0.91) * | 1,815 | 0.56 (0.48, 0.66) * |
|  |  |  |  |  |
| No Moderate Activity | 2,774 | Reference | 2,839 | Reference |
| Moderate Activity | 3,399 | 1.22 (1.08, 1.38) * | 2,877 | 0.95 (0.83, 1.08) |
|  |  |  |  |  |
| No Walking | 1,759 | Reference | 1,491 | Reference |
| Walking | 4,414 | 0.86 (0.75, 0.99) * | 4,225 | 0.96 (0.82, 1.12) |
|  |  |  |  |  |
| Consumed in the Last Week: |  |  |  |  |
| *Instant Noodles* |  |  |  |  |
| No | 2,874 | Reference | 2,490 | Reference |
| Yes | 3,296 | 1.00 (0.88, 1.12) | 3,222 | 1.05 (0.92, 1.21) |
|  |  |  |  |  |
| *Fast Food* |  |  |  |  |
| No | 5,728 | Reference | 5,354 | Reference |
| Yes | 442 | 1.85 (1.41, 2.42) * | 358 | 1.76 (1.35, 2.30) * |
|  |  |  |  |  |
| *Soda* |  |  |  |  |
| No | 5,597 | Reference | 4,790 | Reference |
| Yes | 573 | 1.20 (0.96, 1.49) | 922 | 1.40 (1.17, 1.67) * |
|  |  |  |  |  |
| *Fried Snacks* |  |  |  |  |
| No | 2,358 | Reference | 2,089 | Reference |
| Yes | 3,812 | 1.27 (1.12, 1.44) * | 3,623 | 1.25 (1.08, 1.44) * |
|  |  |  |  |  |
| Mean Number of Days Consumed in the Last Week^f^: |  |  |  |  |
| Instant Noodles | 3,296 | 0.98 (0.93, 1.03) | 3,222 | 0.96 (0.91, 1.02) |
| Fast Food | 442 | 0.99 (0.82, 1.20) | 358 | 1.11 (0.89, 1.39) |
| Soda | 573 | 1.12 (0.97, 1.29) | 922 | 0.93 (0.83, 1.04) |
| Fried Snacks | 3,812 | 1.01 (0.97, 1.04) | 3,623 | 1.02 (0.99, 1.06) |
| Household Level |  |  |  |  |
| Food Expenditures^g^ |  |  |  |  |
| *Rice* |  |  |  |  |
| Lowest | 3,550 | Reference | 3,107 | Reference |
| Highest | 3,192 | 1.03 (0.92, 1.16) | 3,016 | 1.03 (0.90, 1.17) |
|  |  |  |  |  |
| *Cooking oil* |  |  |  |  |
| Lowest | 4,079 | Reference | 3,637 | Reference |
| Highest | 2,664 | 1.37 (1.21, 1.54) * | 2,486 | 1.31 (1.15, 1.50) * |
|  |  |  |  |  |
| Residence |  |  |  |  |
| Rural | 2,840 | Reference | 2,650 | Reference |
| Urban | 3,917 | 1.92 (1.72, 2.15) * | 3,486 | 2.21 (1.92, 2.54) * |
|  |  |  |  |  |
| Wealth |  |  |  |  |
| Lowest | 1,230 | Reference | 1,357 | Reference |
| Second | 1,031 | 1.11 (0.91, 1.36) | 1,129 | 1.20 (0.96, 1.48) |
| Middle | 748 | 1.18 (0.95, 1.47) | 882 | 1.18 (0.93, 1.49) |
| Fourth | 832 | 1.15 (0.93, 1.43) | 949 | 1.28 (1.02, 1.59) * |
| Highest | 911 | 1.25 (1.02, 1.54) * | 952 | 1.82 (1.46, 2.25) * |
|  |  |  |  |  |
| Family Size |  |  |  |  |
| ≤ 4 | 4,320 | Reference | 3,746 | Reference |
| > 4 | 2,437 | 0.92 (0.82, 1.04) | 2,390 | 0.99 (0.86, 1.13) |

CI = confidence interval

^a^ Measured among adults ≥ 40 years old. Defined as waist circumference ≥ 90 cm if male and waist circumference ≥ 80 cm if female

^b^ Odds ratios and confidence intervals are estimated using logistic regression and are weighted to account for the survey design. Models exclude women who are currently pregnant.

^c^ Skilled manual labor combines the following employment sectors: mining, manufacturing, electric, gas, water maintenance, and construction

^d^ Skilled labor combines the following employment sectors: retail and service, transportation

^e^ Defined using the International Physical Activity Questionnaire

^f^ Modeled as a continuous variable, the average number of days consumed is queried if the respondent reported that they consumed item in the last week

^g^ Indicates the household level expenditure on each item as a percentage of the households’ total expenditures on food

* *p* < 0.05
