# Supplementary material for: Risk factors for nutrition-related chronic disease among adults in Indonesia
Source: PLoS One. 2019 Aug 30;14(8):e0221927. doi: 10.1371/journal.pone.0221927 (PMC6716634; doi:10.1371/journal.pone.0221927)
Supplement: S4 Table — (DOCX) [file pone.0221927.s004.docx]

**S4 Table. Univariate Logistic Regression Testing the Association Between Selected Characteristics and Type 2 Diabetes Among Adults in Indonesia, 2014**

|  | **Women^a,b^** | | | **Men^a,b^** | |
| --- | --- | --- | --- | --- | --- |
|  | n | | Odds Ratio  (95% CI) | n | Odds Ratio  (95% CI) |
| Individual Level |  | |  |  |  |
| Age (in years) | |  |  |  |  |
| 19-29 | | 720 | Reference | 624 | Reference |
| 30-39 | | 743 | 2.99 (1.44, 6.20) * | 638 | 3.06 (1.54, 6.09) |
| 40-49 | | 450 | 5.82 (2.90, 11.70) * | 403 | 4.57 (2.27, 9.20) |
| 50-59 | | 603 | 8.99 (4.66, 17.34) * | 327 | 5.31 (2.67, 10.55) |
| ≥ 60 | | 858 | 7.84 (4.09, 15.06) * | 701 | 7.24 (3.87, 13.54) |
|  | |  |  |  |  |
| Education | |  |  |  |  |
| No Education | | 365 | Reference | 103 | Reference |
| Primary | | 1,207 | 1.04 (0.69, 1.56) | 896 | 3.78 (1.29, 11.10) * |
| Junior or Senior | | 867 | 0.88 (0.56, 1.38) | 808 | 3.51 (1.19, 10.37) * |
| University | | 285 | 0.74 (0.40, 1.36) | 264 | 6.41 (2.08, 19.76) * |
|  | |  |  |  |  |
| Marital Status | |  |  |  |  |
| Never Married | | 301 | Reference | 507 | Reference |
| Married | | 2,335 | 2.05 (0.97, 4.33) | 2,027 | 3.26 (1.84, 5.78) * |
| Other | | 736 | 2.46 (0.98, 5.33) | 159 | 1.59 (0.66, 3.85) |
|  | |  |  |  |  |
| Employment | |  |  |  |  |
| Not Working | | 1279 | Reference | 361 | Reference |
| Agriculture-based Labor | | 565 | 0.76 (0.51, 1.13) | 773 | 0.34 (0.21, 0.55) * |
| Skilled Manual Labor^c^ | | 280 | 0.64 (0.36, 1.14) | 491 | 0.35 (0.21, 0.58) * |
| Skilled Labor^d^ | | 1,177 | 0.89 (0.65, 1.22) | 1,022 | 0.63 (0.42, 0.94) * |
|  | |  |  |  |  |
| Overweight/Obese | |  |  |  |  |
| No | | 1,958 | Reference | 2,097 | Reference |
| Yes | | 1,289 | 2.60 (1.96, 3.44) * | 580 | 4.76 (3.44, 6.59) * |
| Smoking Status | |  |  |  |  |
| Does not smoke | | 3,207 | Reference | 921 | Reference |
| Currently Smoking | | 141 | 0.88 (0.48, 1.62) | 1,756 | 0.65 (0.47, 0.89) * |
|  | |  |  |  |  |
| Physical Activity in the Last Week^e^: | |  |  |  |  |
|  | |  |  |  |  |
| No Vigorous Physical Activity | | 2,752 | Reference | 1,581 | Reference |
| Vigorous Physical Activity | | 343 | 0.65 (0.39, 1.07) | 925 | 0.49 (0.33, 0.73) * |
|  | |  |  |  |  |
| No Moderate Physical Activity | | 1,371 | Reference | 1,188 | Reference |
| Moderate Physical Activity | | 1,724 | 0.78 (0.59, 1.03) | 1,318 | 0.65 (0.47, 0.91) * |
|  | |  |  |  |  |
| No Walking | | 970 | Reference | 673 | Reference |
| Walking | | 2,125 | 0.97 (0.71, 1.31) | 1,833 | 0.81 (0.57, 1.15) |
|  | |  |  |  |  |
| Consumed in the Last Week: | |  |  |  |  |
| *Instant Noodles* | |  |  |  |  |
| No | | 1,290 | Reference | 935 | Reference |
| Yes | | 1,804 | 0.73 (0.55, 0.97) * | 1,569 | 0.87 (0.63, 1.22) |
|  | |  |  |  |  |
| *Fast Food* | |  |  |  |  |
| No | | 2,796 | Reference | 2,263 | Reference |
| Yes | | 298 | 1.11 (0.68, 1.80) | 241 | 0.89 (0.51, 1.55) |
|  | |  |  |  |  |
| *Soda* | |  |  |  |  |
| No | | 2,761 | Reference | 1,921 | Reference |
| Yes | | 333 | 0.57 (0.34, 0.96) * | 583 | 0.80 (0.53, 1.20) |
|  | |  |  |  |  |
| *Fried Snacks* | |  |  |  |  |
| No | | 1,174 | Reference | 878 | Reference |
| Yes | | 1,920 | 0.75 (0.57, 1.00) | 1,626 | 0.78 (0.56, 1.09) |
|  | |  |  |  |  |
| Mean Number of Days Consumed in the Last Week^f^: | |  |  |  |  |
| Instant Noodles | | 1,804 | 1.05 (0.94, 1.18) | 1,569 | 1.05 (0.93, 1.19) |
| Fast Food | | 298 | 1.21 (0.96, 1.52) | 241 | 0.38 (0.16, 0.90) * |
| Soda | | 333 | 0.90 (0.68, 1.20) | 583 | 0.89 (0.71, 1.12) |
| Fried Snacks | | 1,920 | 1.04 (0.96, 1.12) | 1,626 | 1.06 (0.97, 1.16) |
| Household Level | |  |  |  |  |
| Food Expenditures^g^ | |  |  |  |  |
| *Rice* | |  |  |  |  |
| Lowest | | 1,875 | Reference | 2,684 | Reference |
| Highest | | 1,491 | 0.97 (0.74, 1.28) | 2,684 | 1.31 (0.96, 1.80) |
|  | |  |  |  |  |
| *Cooking oil* | |  |  |  |  |
| Lowest | | 2,069 | Reference | 2,684 | Reference |
| Highest | | 1,298 | 0.97 (0.74, 1.29) | 2,684 | 1.02 (0.73, 1.42) |
|  | |  |  |  |  |
| Residence | |  |  |  |  |
| Rural | | 1,421 | Reference | 1,123 | Reference |
| Urban | | 1,953 | 1.28 (0.97, 1.69) | 1,569 | 1.49 (1.08, 2.07) * |
|  | |  |  |  |  |
| Wealth | |  |  |  |  |
| Lowest | | 580 | Reference | 560 | Reference |
| Second | | 535 | 1.03 (0.64, 1.64) | 486 | 1.39 (0.83, 2.33) |
| Middle | | 396 | 1.45 (0.86, 2.45) | 319 | 1.16 (0.63, 2.14) |
| Fourth | | 451 | 1.20 (0.73, 1.96) | 410 | 1.48 (0.86, 2.54) |
| Highest | | 449 | 1.12 (0.68, 1.86) | 367 | 1.70 (1.01, 2.88) * |
|  | |  |  |  |  |
| Family Size | |  |  |  |  |
| ≤ 4 | | 2,090 | Reference | 1,661 | Reference |
| > 4 | | 1,284 | 0.82 (0.62, 1.08) | 1,031 | 0.76 (0.54, 1.06) |

CI = confidence interval

^a^ Defined as HbA_1c_ ≥ 6.5%

^b^ Odds ratios and confidence intervals are estimated using logistic regression and are weighted to account for the survey design. Models exclude women who are currently pregnant.

^c^ Skilled manual labor combines the following employment sectors: mining, manufacturing, electric, gas, water maintenance, and construction

^d^ Skilled labor combines the following employment sectors: retail and service, transportation

^e^ Defined using the International Physical Activity Questionnaire

^f^ Modeled as a continuous variable, the average number of days consumed is queried if the respondent reported that they consumed item in the last week

^g^ Indicates the household level expenditure on each item as a percentage of the households’ total expenditures on food

* *p* < 0.05
