# Supplementary material for: Risk factors for nutrition-related chronic disease among adults in Indonesia
Source: PLoS One. 2019 Aug 30;14(8):e0221927. doi: 10.1371/journal.pone.0221927 (PMC6716634; doi:10.1371/journal.pone.0221927)
Supplement: S5 Table — (DOCX) [file pone.0221927.s005.docx]

**S5 Table. Multivariable Logistic Regression Testing the Association Between Selected Characteristics and Hypertension Among Adults in Indonesia, 2014**

|  | **Women^a,b^** | **Men^a,b^** |
| --- | --- | --- |
|  | N=8,854 | N =8,745 |
| Individual Level | Odds Ratio (95% CI) | Odds Ratio (95% CI) |
| Age (in years) |  |  |
| 19-29 | Reference | Reference |
| 30-39 | 2.65 (2.06, 3.42) * | 1.17 (0.87, 1.57) |
| 40-49 | 6.53 (5.06, 8.42) * | 2.53 (1.88, 3.41) * |
| 50-59 | 13.97 (10.68, 18.28) * | 4.28 (3.15, 5.81) * |
| ≥ 60 | 23.51 (17.32, 31.91) * | 9.22 (6.65, 12.79) * |
|  |  |  |
| Education |  |  |
| No Education | Reference |  |
| Primary | 1.08 (0.84, 1.38) | 0.99 (0.68, 1.44) |
| Junior or Senior | 0.84 (0.64, 1.10) | 0.89 (0.59, 1.33) |
| University | 0.66 (0.47, 0.92) * | 0.98 (0.62, 1.55) |
|  |  |  |
| Marital Status |  |  |
| Never Married | Reference | Reference |
| Married | 1.08 (0.67, 1.76) | 0.74 (0.54, 1.03) |
| Other | 1.15 (0.67, 1.97) | 1.08 (0.63, 1.86) |
|  |  |  |
| Employment |  |  |
| Not Working | Reference | Reference |
| Agriculture-based Labor | 0.66 (0.54, 0.79) * | 0.63 (0.47, 0.85) * |
| Skilled Manual Labor^c^ | 0.81 (0.63, 1.04) | 0.69 (0.50, 0.94) * |
| Skilled Labor^d^ | 0.73 (0.62, 0.85) * | 0.69 (0.52, 0.92) * |
|  |  |  |
| **Overweight (BMI ≥ 23 kg/m^2^)** |  |  |
| No | Reference | Reference |
| Yes | 2.00 (1.74, 2.31) * | 2.74 (2.33, 3.21) * |
|  |  |  |
| Smoking Status |  |  |
| Does not smoke | Reference | Reference |
| Currently Smoking | 1.09 (0.75, 1.57) | 0.85 (0.73, 1.00) |
|  |  |  |
| Physical Activity in the Last Week^e^:: |  |  |
|  |  |  |
| No Vigorous Activity |  | Reference |
| Vigorous Activity | --- | 0.82 (0.70, 0.97) * |
|  |  |  |
| No Moderate Activity | Reference | Reference |
| Moderate Activity | 0.95 (0.84, 1.08) | 0.92 (0.79, 1.07) |
|  |  |  |
| Mean Number of Days Consumed in the Last Week^f^ |  |  |
|  |  |  |
| *Instant Noodles* |  |  |
| No | Reference | Reference |
| Yes | 0.99 (0.87, 1.14) | 0.99 (0.85, 1.16) |
|  |  |  |
| *Fast Food* |  |  |
| No | Reference | Reference |
| Yes | 1.06 (0.84, 1.33) | 1.01 (0.75, 1.34) |
|  |  |  |
| *Soda* |  |  |
| No | Reference | Reference |
| Yes | 0.95 (0.77, 1.18) | 1.00 (0.82, 1.22) |
| Household Level |  |  |
| Residence |  |  |
| Rural | Reference | Reference |
| Urban | 1.05 (0.92, 1.22) | 1.00 (0.85, 1.18) |
| Wealth |  |  |
| Lowest | Reference | Reference |
| Second | 0.96 (0.80, 1.16) | 0.79 (0.64, 0.98) * |
| Middle | 1.00 (0.81, 1.22) | 0.87 (0.69, 1.10) |
| Fourth | 0.96 (0.79, 1.17) | 0.81 (0.65, 1.02) |
| Highest | 0.90 (0.74, 1.09) | 0.78 (0.61, 0.99) * |
| Family Size |  |  |
| ≤ 4 | Reference |  |
| > 4 | 0.92 (0.81, 1.05) | --- |

BMI = body mass index, CI = confidence interval

^a^ Systolic and diastolic blood pressure are based on the average of three measurements. Hypertension is defined as systolic blood pressure ≥140 mm or diastolic blood pressure ≥ 90 mm or current use of antihypertensive medication

^b^ Odds ratios and confidence intervals are estimated using logistic regression and are weighted to account for the survey design. Models exclude women who are currently pregnant.

^c^ Skilled manual labor combines the following employment sectors: mining, manufacturing, electric, gas, water maintenance, and construction

^d^ Skilled labor combines the following employment sectors: retail and service, transportation

^e^ Defined using the International Physical Activity Questionnaire

^f^ Modelled as a continuous variable, the average number of days consumed is queried if the respondent reported that they consumed item in the last week (i.e. these models exclude non-consumers).

* *p* < 0.05
