# Supplementary material for: Risk factors for nutrition-related chronic disease among adults in Indonesia
Source: PLoS One. 2019 Aug 30;14(8):e0221927. doi: 10.1371/journal.pone.0221927 (PMC6716634; doi:10.1371/journal.pone.0221927)
Supplement: S6 Table — (DOCX) [file pone.0221927.s006.docx]

**S6 Table. Multivariable Logistic Regression Testing the Association Between Selected Characteristics and Elevated hs-CRP Among Adults in Indonesia, 2014**

|  | **Women^a,b^** | **Men ^a,b^** |
| --- | --- | --- |
|  | N=1,055 | N=453 |
| Individual Level | Odds Ratio (95% CI) | Odds Ratio (95% CI) |
| Age (in years) |  |  |
| 19-29 | --- | Reference |
| 30-39 | --- | 1.33 (0.54, 3.29) |
| 40-49 | --- | 1.13 (0.40, 3.15) |
| 50-59 | --- | 0.58 (0.15, 2.27) |
| ≥ 60 | --- | 1.33 (0.44, 4.00) |
|  |  |  |
| Education |  |  |
| No Education | Reference |  |
| Primary | 1.33 (0.55, 3.25) | --- |
| Junior or Senior | 1.03 (0.42, 2.52) | --- |
| University | 0.81 (0.28, 2.30) | --- |
|  |  |  |
| Marital Status |  |  |
| Never Married | Reference | Reference |
| Married | 2.57 (1.09, 6.07) | 1.50 (0.57, 3.96) |
| Other | 0.57 (0.17, 1.84) | 5.27 (0.80, 34.54) |
|  |  |  |
| Employment |  |  |
| Not Working |  | Reference |
| Agriculture-based Labor | 0.59 (0.32, 1.08) | 1.71 (0.49, 5.99) |
| Skilled Manual Labor^c^ | 0.47 (0.23, 0.96) * | 0.76 (0.20, 2.86) |
| Skilled Labor^d^ | 0.99 (0.65, 1.51) | 1.43 (0.40, 5.06) |
|  |  |  |
| **Overweight (BMI ≥ 23 kg/m^2^)** |  |  |
| No | Reference | Reference |
| Yes | 1.51 (1.03, 2.21) * | 1.63 (0.87, 3.05) |
|  |  |  |
| Physical Activity (Last Week)^e^: |  |  |
| No Vigorous Activity | Reference | Reference |
| Vigorous Activity | 0.71 (0.40, 1.27) | 0.64 (0.36, 1.14) |
|  |  |  |
| Consumed (Last Week): |  |  |
| *Fast Food* |  |  |
| No | Reference |  |
| Yes | 1.06 (0.60, 1.87) | --- |
|  |  |  |
| Mean Number of Days Consumed Last Week^f^: |  |  |
| Instant Noodles | 1.13 (1.02, 1.25) * | --- |
| Soda | --- | 1.31 (1.09, 1.57) * |
| Household Level |  |  |
| Residence |  |  |
| Rural | Reference | Reference |
| Urban | 1.61 (1.06, 2.43) * | 1.49 (0.77, 2.87) |
|  |  |  |
| Wealth |  |  |
| Lowest | Reference | Reference |
| Second | 1.04 (0.61, 1.79) | 1.32 (0.52, 3.38) |
| Middle | 0.85 (0.48, 1.53) | 1.16 (0.40, 3.34) |
| Fourth | 0.93 (0.54, 1.60) | 0.50 (0.19, 1.31) |
| Highest | 0.76 (0.44, 1.32) | 1.31 (0.56, 3.08) |
| BMI = body mass index, CI = confidence interval; hs-CRP = high sensitivity c-reactive protein  ^a^ Defined as hs-CRP > 3 mg/dL  ^b^ Odds ratios and confidence intervals are estimated using logistic regression and are weighted to account for the survey design. Models exclude women who are currently pregnant.  ^c^ Skilled manual labor combines the following employment sectors: mining, manufacturing, electric, gas, water maintenance, and construction  ^d^ Skilled labor combines the following employment sectors: retail and service, transportation  ^e^ Defined using the International Physical Activity Questionnaire  ^f^ Modelled as a continuous variable, the average number of days consumed is queried if the respondent reported that they consumed item in the last week (i.e. these models exclude non-consumers).  * *p* < 0.05 | | |
