# Supplementary material for: Risk factors for nutrition-related chronic disease among adults in Indonesia
Source: PLoS One. 2019 Aug 30;14(8):e0221927. doi: 10.1371/journal.pone.0221927 (PMC6716634; doi:10.1371/journal.pone.0221927)
Supplement: S7 Table — (DOCX) [file pone.0221927.s007.docx]

**S7 Table. Multivariable Logistic Regression Testing the Association Between Selected Characteristics and Type 2 Diabetes Among Adults in Indonesia, 2014**

|  | **Women^a,b^** | **Men^a,b^** |
| --- | --- | --- |
|  | N=2,045 | N=1,596 |
| Individual Level | Odds Ratio (95% CI) | Odds Ratio (95% CI) |
| Age (in years) |  |  |
| 19-29 | Reference | Reference |
| 30-39 | 2.94 (0.98, 8.83) | 4.83 (0.90, 26.03) |
| 40-49 | 4.55 (1.57, 13.18) * | 5.70 (0.83, 39.23) |
| 50-59 | 7.81 (2.79, 21.82) * | 9.26 (1.49, 57.59) * |
| ≥ 60 | 8.53 (3.01, 24.13) * | 16.13 (2.58,100.80) * |
|  |  |  |
| Education |  |  |
| No Education |  | Reference |
| Primary | --- | 4.96 (1.06, 23.14) * |
| Junior or Senior | --- | 5.41 (1.10, 26.69) * |
| University | --- | 6.38 (1.20, 33.87) * |
|  |  |  |
| Marital Status |  |  |
| Never Married |  | Reference |
| Married | --- | 0.62 (0.12, 3.19) |
| Other | --- | 0.63 (0.09, 4.64) |
|  |  |  |
| Employment |  |  |
| Not Working |  | Reference |
| Agriculture-based Labor | --- | 0.51 (0.26, 1.03) |
| Skilled Manual Labor^c^ | --- | 0.55 (0.26, 1.17) |
| Skilled Labor^d^ | --- | 0.93 (0.51, 1.67) |
|  |  |  |
| **Overweight (BMI ≥ 23 kg/m^2^)** |  |  |
| No | Reference | Reference |
| Yes | 2.48 (1.61, 3.83) * | 3.32 (2.17, 5.07) * |
|  |  |  |
| Smoking Status |  |  |
| Does not smoke |  | Reference |
| Currently Smoking | --- | 1.11 (0.73, 1.69) |
|  |  |  |
| Physical Activity (Last Week)^e^: |  |  |
| No Vigorous Activity |  | Reference |
| Vigorous Activity | --- | 0.58 (0.34, 1.00) |
|  |  |  |
| No Moderate Activity |  | Reference |
| Moderate Activity | --- | 0.79 (0.52, 1.20) |
|  |  |  |
| Consumed (Last Week): |  |  |
| *Instant Noodles* |  |  |
| No | Reference |  |
| Yes | 0.93 (0.65, 1.34) | --- |
|  |  |  |
| *Soda* |  |  |
| No | Reference |  |
| Yes | 0.64 (0.33, 1.25) | --- |
| Household Level |  |  |
| Residence |  |  |
| Rural | Reference | Reference |
| Urban | 1.34 (0.94, 1.92) | 0.98 (0.62, 1.56) |
|  |  |  |
| Wealth |  |  |
| Lowest | Reference | Reference |
| Second | 1.05 (0.63, 1.74) | 1.81 (0.99, 3.30) |
| Middle | 1.61 (0.93, 2.81) | 1.19 (0.60, 2.39) |
| Fourth | 1.27 (0.75, 2.16) | 1.46 (0.76, 2.82) |
| Highest | 1.19 (0.68, 2.06) | 1.43 (0.71, 2.87) |
| BMI = body mass index, CI = confidence interval  ^a^ Defined as HbA_1c_ ≥ 6.5%  ^b^ Odds ratios and confidence intervals are estimated using logistic regression and are weighted to account for the survey design. Models exclude women who are currently pregnant.  ^c^ Skilled manual labor combines the following employment sectors: mining, manufacturing, electric, gas, water maintenance, and construction  ^d^ Skilled labor combines the following employment sectors: retail and service, transportation  ^e^ Defined using the International Physical Activity Questionnaire  * *p* < 0.05 | | |
